# Polydatin inhibits ZEB1‐invoked epithelial‐mesenchymal transition in fructose‐induced liver fibrosis

**DOI:** 10.1111/jcmm.15933

**Published:** 2020-10-15

**Authors:** Xiaojuan Zhao, Yanzi Yang, Hanwen Yu, Wenyuan Wu, Yang Sun, Ying Pan, Lingdong Kong

**Affiliations:** ^1^ State Key Laboratory of Pharmaceutical Biotechnology School of Life Sciences Nanjing University Nanjing China

**Keywords:** EMT, excess fructose intake, miR‐203, polydatin, ZEB1

## Abstract

High fructose intake is a risk factor for liver fibrosis. Polydatin is a main constituent of the rhizome of *Polygonum cuspidatum*, which has been used in traditional Chinese medicine to treat liver fibrosis. However, the underlying mechanisms of fructose‐driven liver fibrosis as well as the actions of polydatin are not fully understood. In this study, fructose was found to promote zinc finger E‐box binding homeobox 1 (ZEB1) nuclear translocation, decrease microRNA‐203 (miR‐203) expression, increase survivin, activate transforming growth factor β1 (TGF‐β1)/Smad signalling, down‐regulate E‐cadherin, and up‐regulate fibroblast specific protein 1 (FSP1), vimentin, N‐cadherin and collagen I (COL1A1) in rat livers and BRL‐3A cells, in parallel with fructose‐induced liver fibrosis. Furthermore, ZEB1 nuclear translocation‐mediated miR‐203 low‐expression was found to target survivin to activate TGF‐β1/Smad signalling, causing the EMT in fructose‐exposed BRL‐3A cells. Polydatin antagonized ZEB1 nuclear translocation to up‐regulate miR‐203, subsequently blocked survivin‐activated TGF‐β1/Smad signalling, which were consistent with its protection against fructose‐induced EMT and liver fibrosis. These results suggest that ZEB1 nuclear translocation may play an essential role in fructose‐induced EMT in liver fibrosis by targeting survivin to activate TGF‐β1/Smad signalling. The suppression of ZEB1 nuclear translocation by polydatin may be a novel strategy for attenuating the EMT in liver fibrosis associated with high fructose diet.

## 1 INTRODUCTION

Excessive fructose consumption induces metabolic syndrome, which aggravates the progression of liver fibrosis in patients and animals.^1^, ^2^, ^3^ Lack of effective pharmacotherapy for liver fibrosis largely attributes to an incomplete understanding of the pathogenesis. Epithelial‐mesenchymal transition (EMT) is a biological process in organ fibrosis, characterized by low level of E‐cadherin (an epithelial marker) and high level of fibroblast specific protein 1 (FSP1, a mesenchymal marker).^4^, ^5^ Transforming growth factor‐β1 (TGF‐β1) signalling is one of the most important profibrotic pathways.^5^ TGF‐β1 induces phosphorylation of Smad2 (p‐Smad2) and p‐Smad3, which subsequently recruit their co‐mediator Smad4 to nuclear translocation, and then regulate hepatocytic phenotype and function.^6^, ^7^ Survivin as a crucial inhibitor of apoptosis promotes bile duct ligation‐induced rat liver fibrosis,^8^ positively regulates TGF‐β1 expression in adenoid cystic carcinoma cases^9^ and provokes the EMT in glioblastoma.^10^ Recently, excess fructose consumption is reported to increase collagen content of liver parenchyma in rodents.^3^ In this regard, survivin may activate TGF‐β1/Smad signalling to promote fructose‐caused the EMT process in liver fibrosis.

Of note, survivin/baculoviral IAP repeat containing 5 (BIRC5) is identified as a target gene of microRNA‐203 (miR‐203).^11^ MiR‐203 inhibits the EMT of ovarian cancer cell line by targeting BIRC5 and blocking TGF‐β signal pathway.^12^ Moreover, miR‐203 expression is decreased in hepatitis C virus core protein‐simulated human hepatocyte cell line.^13^ However, it remains unknown how fructose alters miR‐203 expression and whether this event affects survivin‐activated TGF‐β1/Smad signalling in the process of the EMT in liver fibrosis.

Zinc finger E‐box binding homeobox 1 (ZEB1) as a transcription factor suppresses the transcription of miR‐203 in human cancer cells.^14^, ^15^ It up‐regulates survivin gene expression and inhibits E‐cadherin nuclear re‐expression in thyroid papillary carcinoma cell line.^16^ In addition, ZEB1 induces TGF‐β1 expression in dimethylnitrosamine‐induced liver fibrosis of rats.^17^ However, it remains unclear whether ZEB1 nuclear translocation mediates miR‐203 deregulation targeting survivin, which is required for TGF‐β1/smad signalling activation in fructose‐driven hepatocyte EMT.

Polydatin is a major active ingredient derived from the rhizome of *Polygonum cuspidatum* Siebold & Zucc, which alleviates liver fibrosis in patients and experimental animals.^18^, ^19^, ^20^ Previous studies have shown that polydatin down‐regulates TGF‐β1, collagen and p‐Smad3 in diet‐induced fibrotic liver of mice,^21^ up‐regulates E‐cadherin and represses radiation‐induced EMT in lung tissues of mice.^22^ However, whether polydatin inhibits ZEB1 nuclear translocation to augment miR‐203 and block survivin‐activated TGF‐β1/Smad signalling in the alleviation of fructose‐induced EMT and liver fibrosis remains mostly unexplored.

In this study, we found that ZEB1 nuclear translocation was sufficient to decrease miR‐203 expression, this new action as a suitable alternative for targeting survivin‐activated TGF‐β1/Smad signalling in fructose‐driven EMT and liver fibrosis. Additionally, we found that polydatin suppressed ZEB1 nuclear translocation to increase miR‐203 expression, and then down‐regulated survivin to block TGF‐β1/Smad signalling activation, resulting in the alleviation of fructose‐caused EMT and liver fibrosis.

## 2 MATERIALS AND METHODS

### 2.1 Reagents and antibodies

Fructose crystallization (Shandong Xiwang Sager Industry Co., Ltd.), polydatin (purity ≥ 98%) (Nanjing Spring & Autumn Biological Engineering Co., Ltd.) and pioglitazone table (Jiangsu DeYuan Pharmaceutical Co., Ltd.) were used to animal experiments. Fructose, polydatin and pioglitazone used to cell experiments were purchased from Sigma‐Aldrich Inc Triglyceride (TG), alanine aminotransferase (ALT), aspartate aminotransferase (AST), hydroxyproline assay kits, as well as insulin, interleukin‐1β (IL‐1β), tumour necrosis factor‐α (TNF‐α), hyaluronic acid, laminin, type III procollagen and TGF‐β1 enzyme‐linked immunosorbent assay (ELISA) kits were obtained from Jiancheng Biotechnology Co., Ltd. The rat normal hepatocyte line (BRL‐3A) was obtained from Shanghai Institutes for Biological Sciences. The nuclear and cytoplasm protein extraction kits were got from Keygen Biotechnology Co., Ltd. Invitrogen^TM^ TRIzol reagent was obtained from Thermo Fisher Scientific. The reverse transcription system kit and ChamQ SYBR qPCR master mix were got from Vazyme Biotechnology Co., Ltd. The dual‐luciferase reporter assay system kit was obtained from Promega Corporation. MiR‐203 mimic, *ZEB1* siRNA, *survivin* siRNA, *TGF‐β1* siRNA, the respective negative control and GP‐miRGL0 reporter vector listed in Table 1 were provided by GenePharma Co., Ltd. The following antibodies were purchased from commercial sources: anti‐survivin (sc‐10811), anti‐Histone H3 (sc‐517576), anti‐TGF‐β1 (sc‐146), anti‐p‐Smad3 (Ser208, sc‐130218), anti‐Smad3 (sc‐116400), anti‐p‐Smad2 (Ser467, sc‐101801), anti‐Smad2 (sc‐6200), anti‐Smad4 (sc‐7966) and anti‐GAPDH (sc‐25778) (Santa Cruz Biotechnology); anti‐E‐cadherin (20874‐1), anti‐FSP1 (66489‐1) and N‐cadherin (66219‐1) (Proteintech); anti‐ZEB1 (ab203829), anti‐α‐SMA (ab5694) and anti‐COL1A1 (ab34710) (Abcam); anti‐β‐actin (ABM‐0001) (Zoonbio Biotechnology); anti‐Lamin A/C (#4777) (Cell Signaling Technology); HRP‐conjugated rabbit anti‐IgG (#AP132P) (Millipore); anti‐vimentin (AF2105), HRP‐conjugated mouse anti‐IgG (HAF007) and HRP‐conjugated goat anti‐IgG (HAF017) (R&D); Alexa Fluor 488 goat anti‐rabbit IgG (A11008) and Alexa Fluor 555 goat anti‐mouse IgG (A21422) (Life Technologies).

**Table 1:** Sequences used for qRT‐PCR, cell transfection and dual‐luciferase reporter assay

Gene Name	Sence primer (5′ → 3′)	Antisence primer (5′ → 3′)
miR‐203	CGCCGGTGAAATGTTTAGGA	CAGCCACAAAAGAGCACAAT
miR‐203 stem‐loop	CCTGTTGTCTCCAGCCACAAAAGAGCACAATATTTCAGGAGACAACAGGCTAGTGG	
U6	CTCGCTTCGGCAGCACA	AACGCTTCACGAATTTGCGT
URP	TGGTGTCGTGGAGTCG	
miR‐203 mimic	GUGAAAUGUUUAGGACCACUAG	AGUGGUCCUAAACAUUUCACUU
Negative control	UUCUCCGAACGUGUCACGUTT	ACGUGACACGUUCGGAGAATT
r*ZEB1*‐siRNA	CCAGCGGUCAUGAUGACAATT	UUGUCAUCAUGACCGCUGGTT
	GCUCACACAUAAGCAGUAATT	UUACUGCUUAUGUGUGAGCTT
	GCCAACAGUUGGUUUGGUATT	UACCAAACCAACUGUUGGCTT
r*Survivin*‐siRNA	CCGAGAAUGAGCCUGAUUUTT	AAAUCAGGCUCAUUCUCGGTT
	GGAUCUACACCUUCAAGAATT	UUCUUGAAGGUGUAGAUCCTT
	GCGCCUUCCUUACAGUCAATT	UUGACUGUAAGGAAGGCGCTT
r*TGF‐β1*‐siRNA	CUGAGUGGCUGUCUUUUGATT	UCAAAAGACAGCCACUCAGTT
	AGACAUCACACACAGUAUATT	UAUACUGUGUGUGAUGUCUTT
	CAGCUGUACAUUGACUUUATT	UAAAGUCAAUGUACAGCUGTT
rSurvivin	TTGGCTCTTTGTTTGTCCAGTTTCA	GGCTTCATCCACTGCCCTACC
rβ‐actin	TGAGCTGCGTGTGGCCCCTGAG	GGGGCATCGGAACCGCTCATTG
WT Survivin‐3′‐UTR	CTGTTCCTGAGAAAATAAAAAGCCTGTCATTTCAAACACTGCTGTGGACCCTACTGGG	
Mu Survivin‐3′‐UTR	CTGTTCCTGAGAAAATAAAAAGCCTGTGAATACAAACACTGCTGTGGACCCTACTGGG	

Abbreviations: Mu, mutant; r, rat; WT, wild‐type.

### 2.2 Animals and experimental design

**FIGURE 1:**
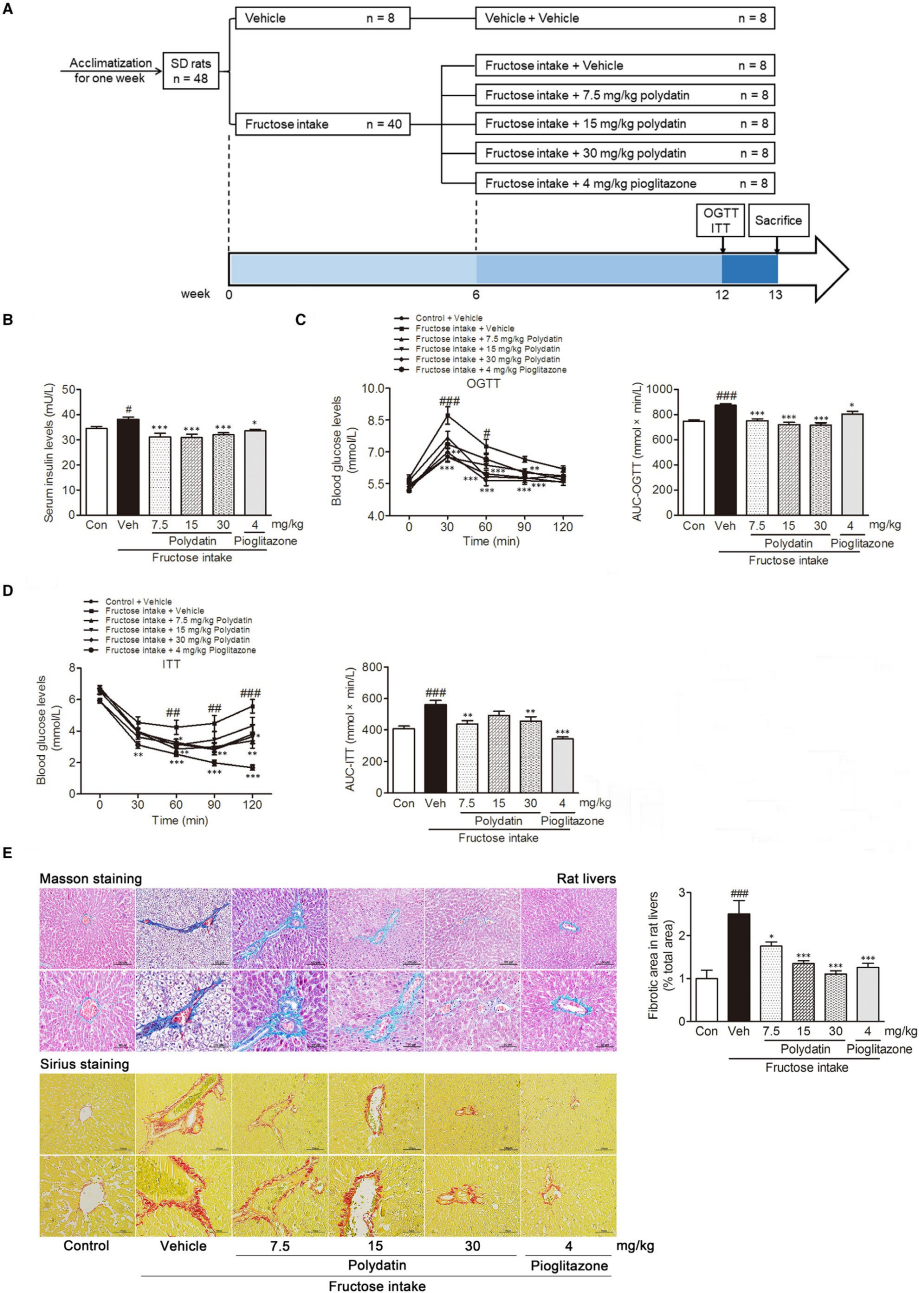
Polydatin alleviates insulin resistance and liver fibrosis in fructose‐fed rats. (A) A schematic representation of the experiments performed and timeline with rats. (B) Serum concentration of insulin was measured. Blood glucose profiles in oral glucose tolerance test (C) and insulin tolerance test (D) were measured, respectively. (E) Representative microphotographs of Masson‐stained and Sirius red‐stained paraffin‐embedded sections of liver tissues were shown (200× magnification: bars, 100 μm; 400× magnification: bars, 50 μm). Fibrotic areas of the Sirius red‐stained liver sections were quantitatively compared. Each value is shown as mean ± SEM (n = 6‐8 in panel A, B and C; n = 3‐4 in panel D). ^#^ *P* < .05, ^##^ *P* < .01, ^###^ *P* < .001 compared with the normal control; *P < .05, **P < .01, ***P < .001 compared with the fructose control

We carried out animal experiments according to the related ethnical regulations of Nanjing University [SYXK (SU) 2009‐0017]. Male Sprague‐Dawley rats (180‐220 g) were provided from Experimental Animal Center of Zhejiang Province (Hangzhou, China; SCXK 2014‐0001). Each rat was given the drinking water or 100 mL drinking water containing 10% fructose (wt/vol) for 6 weeks.^23^, ^24^ Then, rats were randomly divided into six groups (n = 8/group): (a) normal control rats and (b) fructose control rats, which received saline; (c‐e) 7.5, 15 and 30 mg/kg polydatin‐ and fructose‐treated rats; (f) 4 mg/kg pioglitazone (positive drug)‐ and fructose‐treated rats orally for the following 11 weeks. Doses of polydatin and pioglitazone used in this animal experiments were selected based on our previous studies^23^, ^24^ and other reports.^21^, ^22^ A schematic representation of the experiments performed and timeline with rats was provided in Figure 1A.

### 2.3 Oral glucose tolerance test (OGTT) and insulin tolerance test (ITT)

OGTT and ITT were performed as previously described.^25^ Briefly, for OGTT, rats received 1.5 g/kg glucose orally; for ITT, rats were given 0.3 IU/kg insulin intraperitoneally. Then, blood samples were collected from the rat tail veins to test glucose levels with blood glucose metre at 0, 30, 60, 90 and 120 minutes after the treatment of glucose or insulin, respectively.

### 2.4 Serum and tissue collection

At the end of the animal experiments, rats were anaesthetized with 50 mg/kg sodium pentobarbital to collect blood samples from rat carotid artery as well as liver tissues. Blood samples were kept at room temperature for 1 hour and then centrifuged (3000 × *g*, 10 min) to obtain the serum samples for biochemical assays. Some liver samples were fixed with paraformaldehyde for histological study, while the others were stored at −80°C for protein or RNA extraction, biochemical assay, respectively.

### 2.5 Histological study

Liver tissues fixed with paraformaldehyde were embedded in paraffin. Then, liver specimens (4 μm‐thick) were cut and stained with Masson trichrome and Sirius red solution, respectively. These sections were observed and photographed under an optical microscope (Nikon Eclipse Ti‐SR, Nikon), respectively.

### 2.6 Biochemical analysis

Serum concentrations of insulin, IL‐1β and TNF‐α, hyaluronic acid, laminin and type III procollagen were measured with ELISA kits according to the manufactures’ instructions, respectively.

Serum concentrations of TG were measured with commercially available biochemical kit according to the manufactures’ instruction. In brief, 5 μL of serum samples and a serious of standards with different concentrations was added to a 96‐well plate. After 250 μL of working solution was mixed with serum samples and standards, the 96‐well plate was incubated at 37°C for 30 minutes. The absorbance of each well at 510 nm was measured by a microplate reader (Thermo Scientific, Schwerte, Germany). Then, the standard curve was made based on concentrations of standards and the corresponding absorbances. Serum concentrations of TG were calculated with the standard curve, respectively.

Serum activities of AST and ALT were detected with standard diagnostic kits according to the manufactures’ instructions, respectively. Briefly, 20 μL of buffer solution I and 5 μL of serum samples were added to assay wells. At the same time, 20 μL of buffer solution I was added to control wells. Then, these 96‐well plates were incubated at 37°C for 30 minutes. Five microlitres of serum samples was added to control wells, and a serious of standards with different concentrations were injected into standard wells. After 20 μL of buffer solution II addition, the plate was incubated at 37°C for 30 minutes. The reaction in each well was stopped with 200 μL of stop solution. Lastly, the absorbance of each well at 510 nm was tested with the microplate reader. After the standard curve was made, serum activities of AST and ALT were calculated, respectively.

The rat liver tissues were homogenized in 10 wt/vol of sodium chloride on ice and centrifugalized (2500 × *g*, 4°C) for 10 minutes to collect the supernatants. Liver levels of TGF‐β1 and hyaluronic acid, as well as cell culture supernatant concentrations of TGF‐β1 were tested with commercial ELISA kits, respectively.

The liver concentrations of hydroxyproline were detected with standard diagnostic kit according to the manufactures’ instructions. In brief, 100 mg of liver tissues was thoroughly hydrolysed at 95°C for 20 minutes. Three millilitres of hydrolysates and a serious of standards with different concentrations were injected into 10 mL tubes, respectively. After 0.5 mL of solution I, solution II and solution III were sequentially added, these tubes were incubated at 65°C for 5 minutes. Finally, the absorbance of each tube at 550 mm was measured with the microplate reader. After the standard curve was made, liver concentrations of hydroxyproline were calculated, respectively. Protein concentrations of liver samples were detected with BCA protein assay kit and normalized to the data of hydroxyproline levels.

### 2.7 Cell culture

BRL‐3A cells were cultured in DMEM with 10% FBS, 100 U/mL penicillin and 100 mg/mL streptomycin at 37°C in 5% CO_2_. These cells (1.0 × 10^6^ cells/well) were seeded into 6‐well plates for determining nuclear ZEB1 protein levels by Western blot analysis at 0, 0.5, 1, 2, 4 and 6 hours; 12‐well plates (2.5 × 10^5^ cells/well) for detecting miR‐203 expression levels by gene expression analysis at 0, 2.5, 4, 7, 12 and 36 hours, after 5 mM fructose stimulation, respectively.

BRL‐3A cells were treated with or without 5 mM fructose in the presence or absence of polydatin (10, 20 and 40 μM) or pioglitazone (10 μM) in 6‐well plates (1.0 × 10^6^ cells/well) for 1 hour to determine nuclear and cytoplasm ZEB1 protein levels; in 12‐well plates (1.0 × 10^5^ cells/well) for 12 hours to detect miR‐203 expression levels, and for 24 hours to detect survivin mRNA levels; in 6‐well plates (5.0 × 10^5^ cells/well) for 24 hours to determine survivin and TGF‐β1 protein levels; for 48 hours (2.5 × 10^5^ cells/well) to assay p‐Smad2, p‐Smad3, Smad4, E‐cadherin, FSP1, vimentin, N‐cadherin and COL1A1 protein levels, respectively. The concentrations of polydatin and pioglitazone and incubation time were selected based on preliminary experiments.^23^

BRL‐3A cells cultured with 5 mM fructose were transiently transfected with 50 nM *ZEB1* siRNA or negative control (NC) in the presence or absence of 40 μM polydatin or 10 μM pioglitazone for 12 hours to determine miR‐203 expression levels. BRL‐3A cells simulated with 5 mM fructose were transfected with 50 nM miR‐203 mimic or NC in the presence or absence of 40 μM polydatin or 10 μM pioglitazone for 1 hour to detect nuclear ZEB1 protein levels, for 24 hours to detect survivin mRNA and protein levels. Under 5 mM fructose exposure condition, BRL‐3A cells transfected with 50 nM *survivin* siRNA or NC were treated with or without 40 μM polydatin or 10 μM pioglitazone for 12 hours to determine miR‐203 expression levels, for 24 hours to assay TGF‐β1 protein levels. Under 5 mM fructose exposure condition, BRL‐3A cells transfected with 50 nM *TGF‐β1* siRNA or NC were treated with or without 40 μM polydatin or 10 μM pioglitazone for 24 hours to test TGF‐β1 protein levels, for 48 hours to assay p‐Smad2, p‐Smad3, Smad4, E‐cadherin, FSP1, vimentin, N‐cadherin and COL1A1 protein levels, respectively.

### 2.8 Dual‐luciferase reporter assay

Twenty four hours prior, BRL‐3A cells were transiently cotransfected with 50 nM of NC or miR‐203 mimic, and 350 ng of wild‐type or mutant of GP‐miRGL0‐survivin‐3′ UTR vector using the Lipofectamine^TM^ 2000. The Renilla and firefly luciferase were assayed with a dual‐luciferase reporter assay system. Briefly, these cells were harvested and lysed after incubation for 24 hours. Ten microlitres of cells lysate was added to a black flat‐bottomed 96‐well plate. Followed by 100 μL of working solution addition, firefly luciferase signal was tested at 640 nm. The reaction in each well was stopped with 100 μL of stop reagent to detect Renilla luciferase signal at 480 nm. Relative luciferase activity was quantified as the ratio of firefly luciferase signal to Renilla luciferase signal.

### 2.9 Immunofluorescence

BRL‐3A cells were fixed with 4% paraformaldehyde and blocked with immunostainings blocking buffer with 0.5% Triton X‐100. Then, these cells were incubated with anti‐E‐cadherin (1:100), anti‐FSP1 (1:200), anti‐COL1A1 (1:500) and anti‐ZEB1 (1:200) primary antibodies at 4°C overnight, respectively. Expression levels of E‐cadherin and COL1A1 were detected with Alexa Fluor 488 (1:500) secondary antibody, of FSP1 and ZEB1 were tested with Alexa Fluor 555 (1:500) secondary antibody. The nucleus was stained with DAPI. Image was examined under a confocal laser scanning microscope (Leica TCS SP8‐MaiTai MP; Leica, Wetzlar, Germany).

### 2.10 Gene expression analysis

Total RNA was extracted from rat liver tissues and the cultured BRL‐3A cells using TRIzol reagent for analysis of survivin mRNA levels and miR‐203 expression levels. For survivin, reverse transcription was performed with the first‐strand cDNA synthesis kit. The reverse transcription reaction of miR‐203 was performed using the specific stem‐loop RT primers listed in Table 1. The resultant cDNA was used as a template to carry out qRT‐PCR with ChamQ SYBR qPCR master mix kit. Survivin expression was normalized to β‐actin expression, while miR‐203 expression was normalized to U6 expression. The relative transcriptional fold change was calculated as 2^−ΔΔT^. These primers of survivin, β‐actin, miR‐203 and U6 listed in Table 1 were provided by Generay Biotechnology Co., Ltd.

### 2.11 Western blot analysis

The whole, nuclear or cytoplasm proteins from rat liver tissues or the cultured BRL‐3A cells were extracted using lysis buffer, followed by centrifugation at 12 000 g for 15 minutes to collect the supernatant, respectively. The supernatant was quantified with a BCA protein assay kit. Then, the concentrations of all protein samples were adjusted to 1 mg/mL. Protein samples (20 μg) were separated on a 10% SDS‐PAGE and transferred onto nitrocellulose membranes and then blocked with 5% skimmed milk. The proteins were incubated with primary antibodies including anti‐ZEB1 (1:1,000), anti‐Histone H3 (1:500), anti‐Lamin A/C (1:1000), anti‐survivin (1:500), anti‐TGF‐β1 (1:500), anti‐p‐Smad2 (1:800), anti‐Smad2 (1:1000), anti‐p‐Smad3 (1:800), anti‐Smad3 (1:1000), anti‐Smad4 (1:1000), anti‐E‐cadherin (1:2000), anti‐FSP1 (1:2000), anti‐N‐cadherin (1:1000), anti‐vimentin (1:1000), anti‐α‐SMA (1:400), anti‐COL1A1 (1:10 000), anti‐GAPDH (1:2000) and anti‐β‐actin (1:5000), respectively. Next, the secondary antibodies were incubated with the blots and visualized with enhanced chemiluminescence.

### 2.12 Statistical analysis

Data are presented as mean ± standard error of the mean (SEM). Comparisons between two groups were performed by Student's *t* test. ANOVA with further analysed by post hoc Dunnett's test was used for comparison between more than two different groups. *P* < .05 was considered to be significant.

## 3 RESULTS

### 3.1 Polydatin ameliorates liver fibrosis in fructose‐fed rats with metabolic syndrome

First, we examined whether polydatin ameliorated liver fibrosis in fructose‐induced metabolic syndrome of rats. The data from biochemical analysis showed that polydatin significantly decreased serum concentrations of insulin (Figure 1B), TG, IL‐1β and TNF‐α (Table 2), and alleviated insulin resistance in OGTT and ITT (Figure 1C,D) in fructose‐fed rats. Simultaneously, polydatin remarkably alleviated liver histologic changes including slight thickening of the central venous wall, perisinusoidal or portal/peripotal fibrosis and reduced fibrotic area in fructose‐fed rats (Figure 1E). In parallel, it significantly reduced serum levels of hyaluronic acid, laminin and type III procollagen, as well as liver levels of hyaluronic acid and hydroxyproline in this animal model (Table 2). Accordantly, polydatin remarkably decreased serum activities of ALT and AST in fructose‐fed rats (Table 2). Pioglitazone exerted similar effects in this animal model (Table 2 and Figure 1). These data suggest that polydatin and pioglitazone alleviate liver fibrosis to recover liver function in fructose‐fed rats with metabolic syndrome.

**Table 2:** Effects of polydatin on serum and liver parameters in fructose‐fed rats

Group	Control + Vehicle	Fructose + Vehicle	Fructose + Polydatin			Fructose + Pioglizatioon
			7.5 mg/kg	15 mg/kg	30 mg/kg	4 mg/kg
Serum levels						
TG (mmol/L)	0.30 ± 0.02	1.05 ± 0.11^##^, ^**^	0.74 ± 0.09	0.53 ± 0.08^**^	0.47 ± 0.12^**^	0.50 ± 0.15^**^
IL‐1β (pg/mL)	12.42 ± 0.56	15.34 ± 0.22^##^, ^**^	13.86 ± 0.23	13.11 ± 0.36^**^	13.70 ± 0.30^*^	13.60 ± 0.69^*^
TNF‐α (pg/mL)	203.40 ± 3.20	223.64 ± 1.54^#^	203.09 ± 6.22^*^	201.58 ± 4.03^**^	196.11 ± 3.94^***^	204.71 ± 7.85^*^
HA (ng/mL)	1.18 ± 0.09	1.61 ± 0.12^#^	1.65 ± 0.06	1.26 ± 0.14	1.17 ± 0.03^*^	1.80 ± 0.06
Laminin (ng/mL)	646.19 ± 67.73	978.9 ± 84.58^#^	747.20 ± 74.82	533.16 ± 117.60^**^	635.74 ± 100.80^*^	925.91 ± 57.51
III procollogen (ng/mL)	1.76 ± 0.43	10.46 ± 1.96^#^, ^*^	5.70 ± 1.34	4.31 ± 2.28^*^	2.08 ± 0.57^**^	1.00 ± 0.49^***^
ALT (IU/L)	0.71 ± 0.03	20.22 ± 2.21^###^, ^***^	17.09 ± 4.07	11.90 ± 2.40	9.94 ± 2.62^*^	9.32 ± 3.87^*^
AST (IU/L)	7.67 ± 0.37	19.46 ± 3.12^#^, ^*^	7.11 ± 0.25^***^	11.67 ± 1.93^*^	9.91 ± 1.83^**^	11.75 ± 2.11^*^
Liver levels						
HA (pg/mg protein)	9.90 ± 0.60	15.55 ± 0.57^#^, ^*^	14.21 ± 1.06	14.54 ± 0.40	12.63 ± 0.43*	12.99 ± 0.62
Hyp (μg/g protein)	265.11 ± 14.94	329.45 ± 11.02^#^, ^*^	237.48 ± 8.02^***^	238.34 ± 6.59^***^	185.23 ± 14.34^***^	218.73 ± 10.22^***^

Note

Each value is given as the mean ± SEM. (n = 6‐8).

Abbreviations: ALT, alanine aminotransferase; AST, aspartate aminotransferase; HA, hyaluronic acid; Hyp, hydroxyproline; IL‐1β, interleukin‐1β; TG, triglyceride; TNF‐α, tumour necrosis factor‐α.

^#^ *P* < .05.

^##^ *P* < .001.

^###^ *P* < .001 vs the normal control.

^*^ *P* < .05.

^**^ *P* < .01.

^***^ *P* < .001 vs the fructose control.

### 3.2 Polydatin attenuates fructose‐induced EMT in rat liver fibrosis and BRL‐3A cells

**FIGURE 2:**
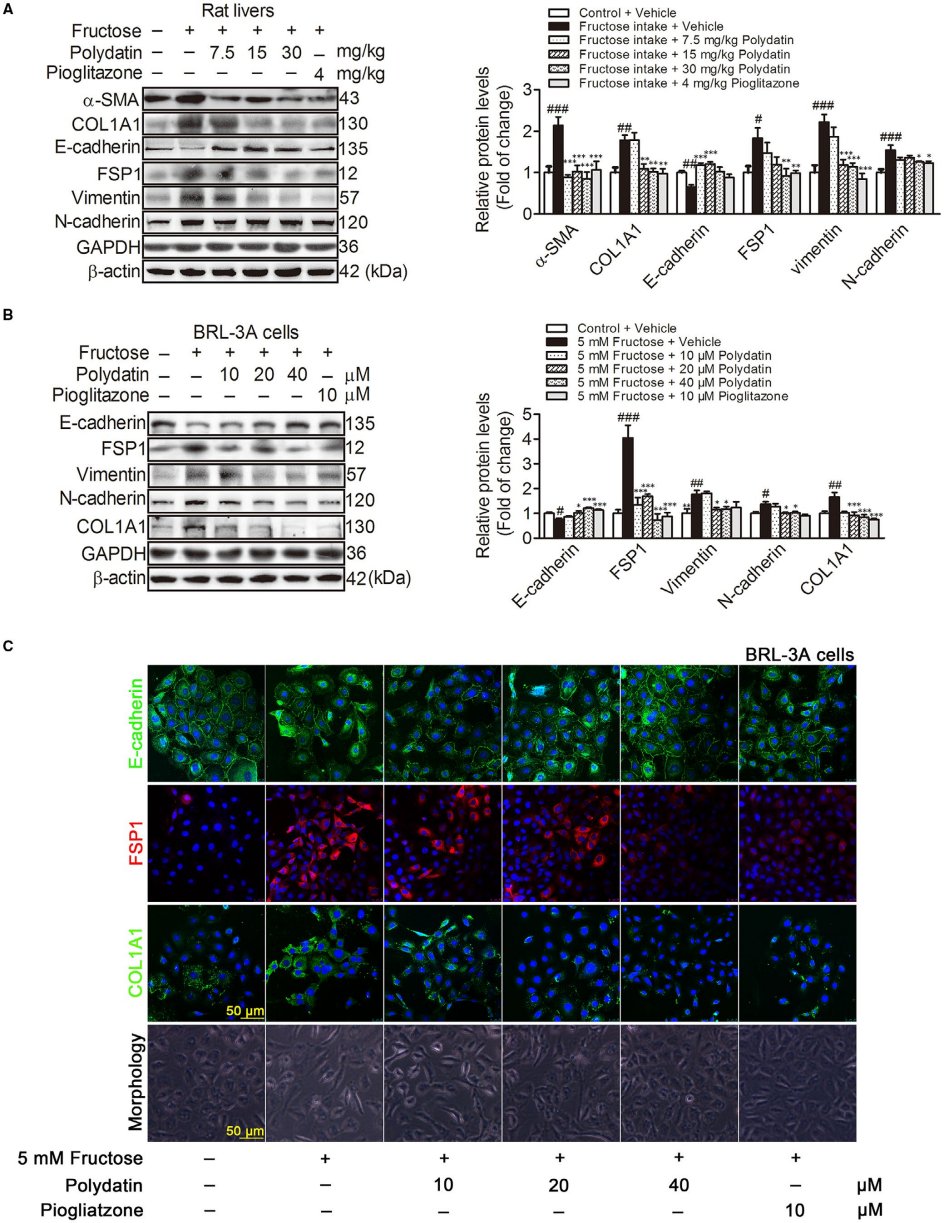
Polydatin attenuates fructose‐induced EMT in liver fibrosis of rats and BRL‐3A cells. (A) Western blot analysis of α‐SMA, COL1A1, E‐cadherin, FSP1, vimentin and N‐cadherin protein levels in rat livers. (B) Western blot analysis of E‐cadherin, FSP1, vimentin, N‐cadherin and COL1A1 protein levels (48 h) in BRL‐3A cells cultured with or without 5 mM fructose in the presence or absence of polydatin (10, 20 and 40 μM) or pioglitazone (10 μM). GAPDH and β‐actin were as internal control. (C) Images of BRL‐3A cells labelled with antibodies specific for E‐cadherin (green), FSP1 (red), COL1A1 (green) as well as morphology. Each value is shown as mean ± SEM (n = 4‐6). ^#^ *P* < .05, ^##^ *P* < .01, ^###^ *P* < .001 compared with the normal control; *P < .05, **P < .01, ***P < .001 compared with the fructose control. FSP1, fibroblast specific protein l; α‐SMA, α smooth muscle actin; COL1A1, collagen 1

EMT plays an important role in liver fibrosis.^5^ Next, we investigated whether polydatin attenuated fructose‐induced EMT in liver fibrosis. As noted previously, polydatin effectively decreased α‐SMA and COL1A1 (fibrotic matrix component), increased E‐cadherin (an epithelial marker), down‐regulated FSP1, vimentin and N‐cadherin (mesenchymal markers) protein levels in fructose‐fed rat livers (Figure 2A). It also increased E‐cadherin, decreased FSP1, vimentin, N‐cadherin and COL1A1 protein levels, and retained epithelial honeycomb‐like morphology in fructose‐exposed BRL‐3A cells (Figure 2B,C). Immunofluorescence staining showed that polydatin reversed fructose‐induced the expression abnormality of E‐cadherin, FSP1 and COL1A1 in BRL‐3A cells (Figure 2C). Pioglitazone had similar actions in these animals and cell models (Figure 2). These results indicate that polydatin and pioglitazone attenuate fructose‐induced EMT in rat liver fibrosis and BRL‐3A cells.

### 3.3 Polydatin suppresses TGF‐β1/Smad signalling activation to attenuate fructose‐caused EMT in liver fibrosis

**FIGURE 3:**
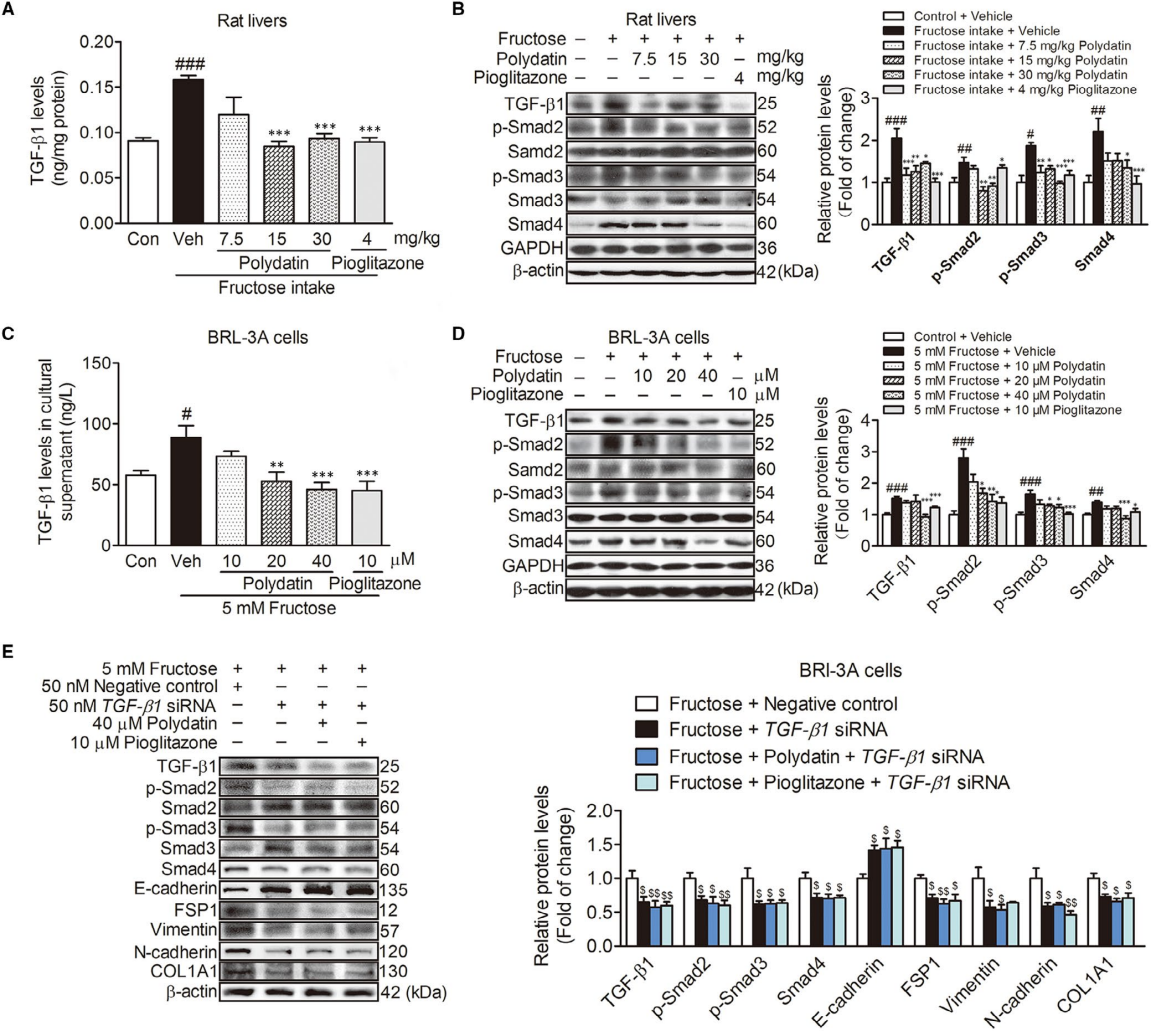
Polydatin suppresses the activation of TGF‐β1/Smad signalling to attenuate fructose‐induced EMT. (A) The liver concentration of TGF‐β1 in the rats was measured with ELISA kit. (B) Western blot analysis of TGF‐β1, p‐Smad2, p‐Smad3 and Smad4 protein levels. (C) TGF‐β1 secretion levels in BRL‐3A cell supernatant were detected with ELISA kit. (D) Western blot analysis of TGF‐β1, p‐Smad2, p‐Smad3 and Smad4 protein levels in BRL‐3A cells (48 h). (E) Western blot analysis of TGF‐β1 (24 h), p‐Smad2, p‐Smad3, Smad4, E‐cadherin, FSP1, vimentin, N‐cadherin and COL1A1 (48 h) protein levels in transfected with 50 nM *TGF‐β1* siRNA or NC BRL‐3A cells treated with fructose in the presence or absence of 40 μM polydatin or 10 μM pioglitazone. GAPDH and β‐actin were as internal control. Each value is shown as mean ± SEM (n = 4‐6). ^#^ *P* < .05, ^##^ *P* < .01, ^###^ *P* < .001 compared with the normal control; *P < .05, **P < .01, ***P < .001 compared with the fructose control; ^$^ *P* < .05, ^$$^ *P* < .01 compared with the fructose‐negative control

TGF‐β1/Smad signalling activation may induce cell towards EMT.^6^, ^7^ Therefore, TGF‐β1 secretion levels, TGF‐β1 as well as its down‐stream protein levels were determined with ELISA or Western blot analysis in rat livers and BRL‐3A cells, respectively. Fructose intake increased TGF‐β1 secretion levels in rat livers (Figure 3A). Protein levels of TGF‐β1, p‐Smad2, p‐Smad3 and Smad4 were also increased in fructose‐fed rat livers (Figure 3B). Consistently, TGF‐β1 secretion was increased in fructose‐exposed BRL‐3A cell supernatants (Figure 3C). TGF‐β1 (24 h), p‐Smad2, p‐Smad3 and Smad4 (48 h) protein levels were increased in fructose‐exposed BRL‐3A cells (Figure 3D), indicating that TGF‐β1/Smad signalling was activated. To address that the activation of TGF‐β1/Smad signalling participated in fructose‐caused EMT, we transfected 50 nM *TGF‐β1* siRNA into BRL‐3A cells treated with fructose and found that *TGF‐β1* siRNA significantly decreased protein levels of TGF‐β1 (24 h), p‐Smad2, p‐Smad3 and Smad4 (48 h) (Figure 3E). Importantly, *TGF‐β1* siRNA significantly increased E‐cadherin, and decreased FSP1, vimentin, N‐cadherin and COL1A1 protein levels in fructose‐exposed BRL‐3A cells (48 h) (Figure 3E). These data indicate that fructose‐induced the activation of TGF‐β1/Smad signalling may mediate the EMT.

Next, we investigated the effect of polydatin on TGF‐β1/Smad signalling activation in fructose‐induced EMT. Polydatin was found to effectively suppress TGF‐β1/Smad signalling activation in fructose‐fed rat livers (Figure 3A,B) and fructose‐exposed BRL‐3A cells (Figure 3C,D). In *TGF‐β1* siRNA‐transfected BRL‐3A cells co‐cultured with fructose, polydatin significantly decreased TGF‐β1 (24 h), p‐Smad2, p‐Smad3 and Smad4 protein levels (48 h), increased E‐cadherin, and decreased FSP‐1, vimentin, N‐cadherin and COL1A1 protein levels (48 h) (Figure 3E). Pioglitazone had similar effects in these animals and cell models (Figure 3). These results indicate that polydatin and pioglitazone alleviate fructose‐caused EMT in liver fibrosis by suppressing the activation of TGF‐β1/Smad signalling.

### 3.4 Polydatin augments miR‐203 targeting survivin to inhibit TGF‐β1/Smad signalling activation

**FIGURE 4:**
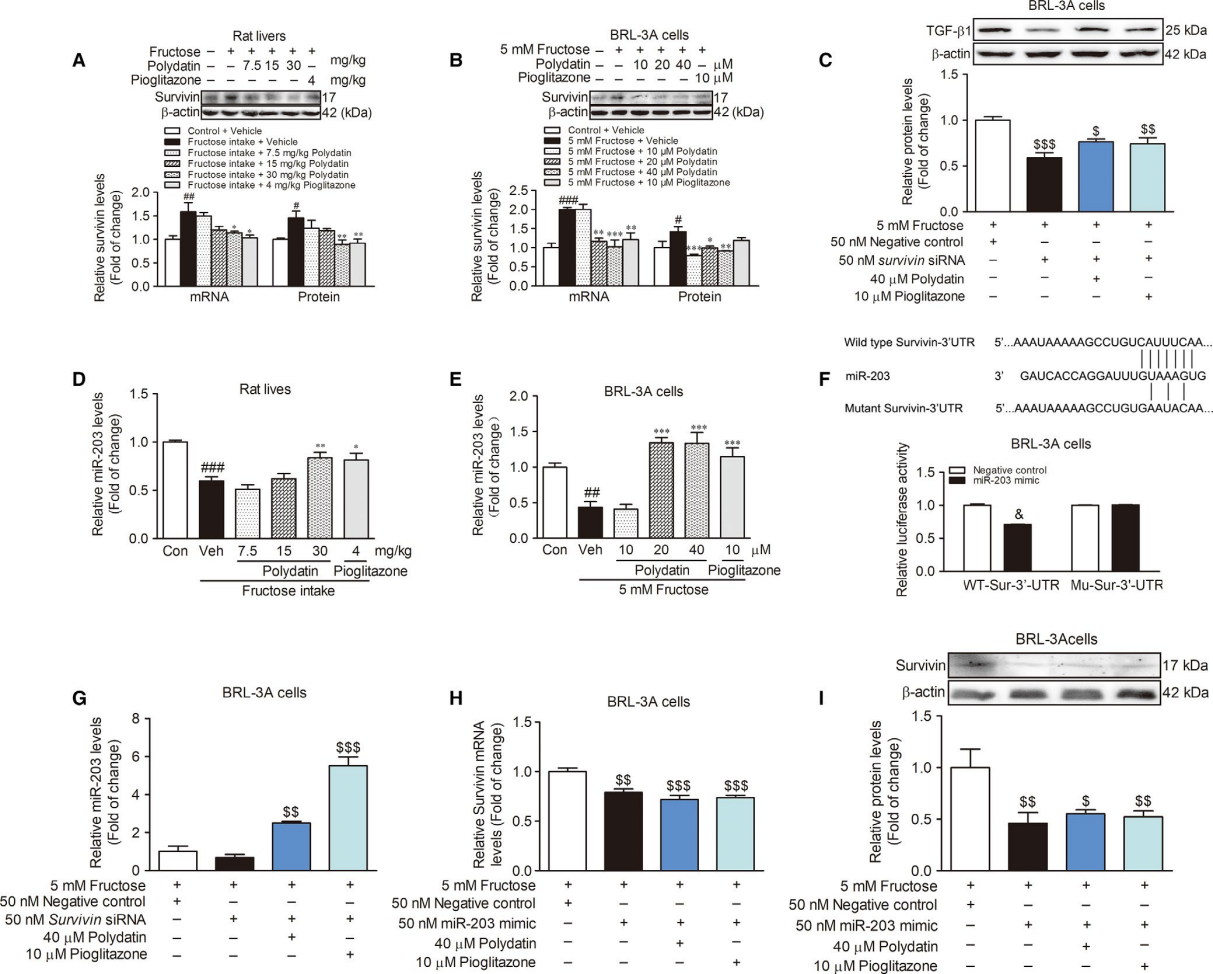
Polydatin augments miR‐203 targeting survivin to inhibit the activation of TGF‐β1/Smad signalling. qRT‐PCR analysis of survivin mRNA levels and Western blot analysis of survivin protein levels in rat livers (A) and in BRL‐3A cells (24 h) (B). (C) TGF‐β1 protein levels (24 h) in transfected with 50 nM *survivin* siRNA or NC BRL‐3A cells exposed to fructose in the presence or absence of 40 μM polydatin or 10 μM pioglitazone. qRT‐PCR analysis of miR‐203 expression in rat livers (D) and BRL‐3A cells (12 h) (E). (F) The assay of survivin 3′‐UTR luciferase activity. (G) MiR‐203 expression in transfected with 50 nM *survivin* siRNA or NC BRL‐3A cells treated with fructose in the presence or absence of 40 μM polydatin or 10 μM pioglitazone. U6 was as internal control. Survivin mRNA (H) and protein (I) levels in transfected with 50 nM miR‐203 mimic or NC BRL‐3A cells co‐cultured with fructose in the presence or absence of 40 μM polydatin or 10 μM pioglitazone. β‐actin was as internal control. Each value is shown as mean ± SEM (n = 4‐6). ^#^ *P* < .05, ^##^ *P* < .01, ^###^ *P* < .001 compared with the normal control; *P < .05, **P < .01, ***P < .001 compared with the fructose control; ^$^ *P* < .05, ^$$^ *P* < .01, ^$$$^ *P* < .001 compared with the fructose‐negative control; ^&^ *P* < .05 compared with the negative control. UTR, untranslated region

Survivin is reported to positively regulate TGF‐β1 expression in adenoid cystic carcinoma cases^9^ and provoke the EMT in glioblastoma.^11^, ^12^ Therefore, we investigated the role of survivin in the activation of TGF‐β1/Smad signalling in fructose‐induced EMT. First, we found that fructose up‐regulated survivin mRNA and protein levels in rat livers and BRL‐3A cells (Figure 4A,B). Then, we transfected BRL‐3A cells with 50 nM *survivin* siRNA and found that *survivin* siRNA significantly blocked fructose‐induced up‐regulation of TGF‐β1 protein levels (24 h) in BRL‐3A cells (Figure 4C).

It is worth noticing that miR‐203 inhibits the EMT of ovarian cancer cell line by targeting survivin/BIRC5 and blocking TGF‐β signal pathway.^12^ miR‐203 is low‐expression in carbon tetrachloride‐induced rat liver fibrosis.^26^ Therefore, we validated miR‐203 expression change in fructose‐caused liver fibrosis. In this study, we found that fructose significantly decreased miR‐203 expression levels in rat livers (Figure 4D) and BRL‐3A cells (Figure 4E). To investigate whether miR‐203 changed survivin expression, we carried out the luciferase assay. The results showed that the luciferase activity in BRL‐3A cells cotransfected with the wild‐type survivin 3′‐UTR reporter vector and miR‐203 mimic was lower than that in the cells transfected with the wild‐type survivin 3′‐UTR reporter and NC (Figure 4F). While the luciferase activity in BRL‐3A cells cotransfected with the mutant survivin 3′‐UTR reporter vector and miR‐203 mimic or NC did not show a significant difference (Figure 4F). Additionally, *survivin* siRNA failed to alter miR‐203 expression (12 h) in BRL‐3A cells (Figure 4G). These data indicate that miR‐203 may target survivin in BRL‐3A cells. To address the regulation of miR‐203 on survivin in fructose‐exposed BRL‐3A cells, we transfected miR‐203 mimic into BRL‐3A cells and observed that miR‐203 mimic significantly abrogated fructose‐induced increase of survivin mRNA and protein levels (24 h) in BRL‐3A cells (Figure 4H,I), indicating that fructose reduced miR‐203 expression to increase survivin.

Furthermore, we observed that polydatin significantly increased miR‐203 expression (Figure 4D,E), and down‐regulated survivin mRNA and protein levels (Figure 4A,B) in fructose‐fed rat livers and fructose‐exposed BRL‐3A cells. Polydatin reversed the effect of *survivin* siRNA to significantly increase miR‐203 expression in fructose‐exposed BRL‐3A cells (Figure 4G). Of note, polydatin markedly decreased survivin mRNA and protein levels (24 h) in miR‐203 mimic‐transfected BRL‐3A cells under fructose exposure (Figure 4H,I). In addition, polydatin down‐regulated TGF‐β1 protein levels (24 h) in fructose‐exposed BRL‐3A cells transfected with *survivin* siRNA (Figure 4C). Pioglitazone had similar effects in these animals and cell models (Figure 4). These results demonstrate that polydatin and pioglitazone augment miR‐203 to down‐regulate survivin, then inhibit TGF‐β1/Smad signalling activation.

### 3.5 Polydatin inhibits ZEB1 nuclear translocation to enhance miR‐203 expression

**FIGURE 5:**
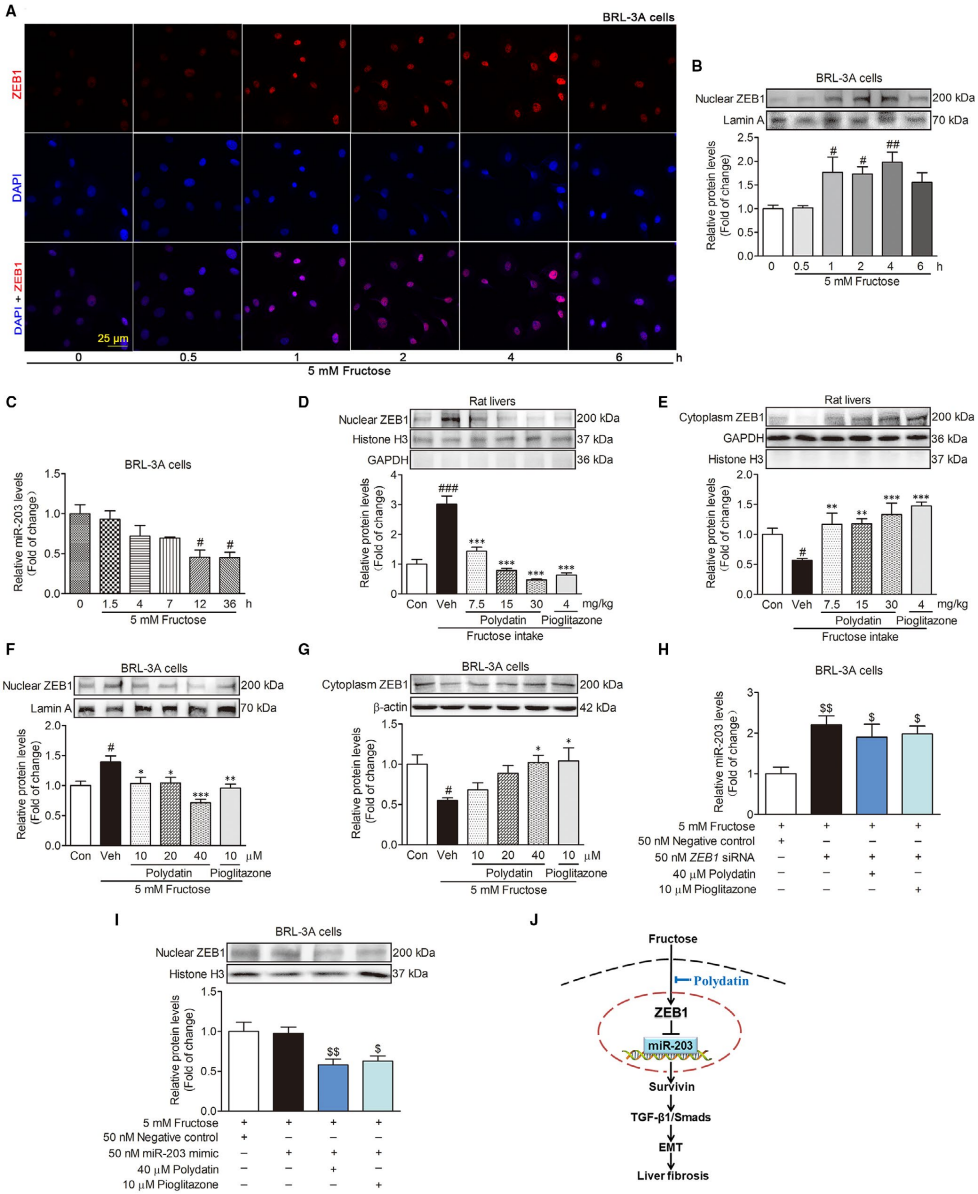
Polydatin inhibits ZEB1 nuclear translocation to enhance miR‐203 expression in fructose‐exposed BRL‐3A cells. (A) Images of fructose‐exposed BRL‐3A cells labelled with ZEB1 (red) at the indicated time points. (B) Western blot analysis of the nuclear ZEB1 protein levels at the indicated time points. (C) qRT‐PCR analysis of miR‐203 expression levels at the indicated time points. Western blot analysis of the nuclear and cytoplasm ZEB1 protein levels in rat livers (D and E) and BRL‐3A cells (2 h) (F and G). (H) qRT‐PCR analysis for expression of miR‐203 (12 h) in transfected with 50 nM *ZEB1* siRNA or NC BRL‐3A cells treated with fructose in the presence or absence of 40 μM polydatin or 10 μM pioglitazone. (I) Nuclear ZEB1 protein levels in transfected with 50 nM miR‐203 mimic or NC BRL‐3A cells exposed to fructose in the presence or absence of 40 μM polydatin or 10 μM pioglitazone. (J) The mechanisms by which polydatin prevents fructose‐induced hepatocyte EMT in liver fibrosis. Histone H3 or Lamin A was as internal control for nuclear ZEB1. β‐actin or GAPDH was as internal control for cytoplasm ZEB1. U6 was as internal control for miR‐203. Each value is shown as mean ± SEM (n = 4‐6). ^#^ *P* < .05, ^##^ *P* < .01, ^###^ *P* < .001 compared with the normal control; *P < .05, **P < .01, ***P < .001 compared with the fructose control; ^$^ *P* < .05, ^$$^ *P* < .01 compared with the fructose‐negative control

ZEB1 as a transcription factor suppresses the transcription of miR‐203 in human cancer cells,^14^, ^15^ thus, we subsequently evaluated the effect of ZEB1 on miR‐203 low expression in fructose‐induced liver fibrosis. We observed significant increase of nuclear ZEB1 protein levels in BRL‐3A cells just 1 hour after fructose exposure (Figure 5A,B). While, miR‐203 expression did not change in response to fructose simulation in BRL‐3A cells during 0‐6 hours (Figure 5C). In fact, fructose intake increased the nuclear ZEB1 protein levels and decreased the cytoplasm ZEB1 protein levels in rat livers (Figure 5D,E). To investigate the role of ZEB1 in the change of miR‐203 expression, we transfected *ZEB1* siRNA into BRL‐3A cells and found that *ZEB1* siRNA significantly induced high‐expression of miR‐203 (12 h) in fructose‐exposed BRL‐3A cells (Figure 5H). While miR‐203 mimic was unable to affect the nuclear ZEB1 protein levels (1 h) in fructose‐exposed BRL‐3A cells (Figure 5I). These data indicate that fructose may cause ZEB1 nuclear translocation to reduce miR‐203 expression, leading to survivin‐mediated the activation of TGF‐β1/Smad signalling in EMT and liver fibrosis.

More importantly, we found that polydatin was able to decrease the nuclear ZEB1 protein levels and increase cytoplasm ZEB1 protein levels in fructose‐fed rat livers (Figure 5D,E) and fructose‐simulated BRL‐3A cells (Figure 5F,G). Polydatin increased miR‐203 expression (12 h) in BRL‐3A cells transfected with *ZEB1* siRNA under fructose exposure condition (Figure 5H). It was able to significantly decrease nuclear ZEB1 protein levels (1 h) in miR‐203 mimic‐transfected BRL‐3A cells co‐cultured with fructose (Figure 5I). Pioglitazone had similar effects in these animals and cell models (Figure 5). These results suggest that polydatin and pioglitazone may inhibit ZEB1 nuclear translocation to enhance miR‐203 expression and then block survivin‐activated TGF‐β1/Smad signalling in fructose‐induced EMT and liver fibrosis.

## 4 DISCUSSION

Clinically, excess fructose consumption is associated with the development of liver fibrosis.^2^ Polydatin is a main constituent in *P cuspidatum*, which has potential utility in the treatment of liver fibrosis in patients.^20^ To the best of our knowledge, we firstly find that ZEB1 nuclear translocation plays an essential role in fructose‐induced EMT in liver fibrosis by targeting survivin to activate TGF‐β1/Smad signalling. Moreover, polydatin represses ZEB1 nuclear translocation to increase miR‐203 expression, subsequently, block survivin‐activated TGF‐β1/Smad signalling, attenuating fructose‐induced EMT in liver fibrosis (Figure 5J).

Generally, activated hepatic stellate cells are considered to be the main source of the extracellular matrix.^27^ While, hepatocytes simulated by TGF‐β1 not only lost the epithelial phenotype with the decrease of E‐cadherin, and the increase of N‐cadherin, vimentin and FSP1, but also produce the collagen,^4^, ^6^, ^7^ indicating that these hepatocytes undergoing EMT induced by TGF‐β1 could be another source of the extracellular matrix. Of note, long‐term fructose intake causes massive collagen deposition of liver parenchyma in the cynomolgus monkeys.^3^ In this study, we showed that fructose triggered EMT in hepatocytes, consistently, it caused rat liver fibrosis. These observations further demonstrated that fructose‐induced hepatocyte EMT, at least to some extent, promoted liver fibrosis process.

In this study, we observed the decrease of E‐cadherin with a relocation of E‐cadherin from the membrane to the cytoplasm in fructose‐exposed BRL‐3A cells. β‐catenin is reported to guide E‐cadherin localization to the cell membrane in Madin‐Darby canine kidney cells.^28^ While, excessive fructose intake decreases β‐catenin protein levels in fibrotic livers of mice.^29^ Thus, we speculated that fructose‐induced β‐catenin reduction may obstruct the localization process of E‐cadherin from the cytoplasm to the cell membrane, which may cause a relocation of E‐cadherin from the membrane to the cytoplasm in fructose‐exposed BRL‐3A cells.

Of note, survivin positively regulates TGF‐β1 in human adenoid cystic carcinoma cell line^9^ and promotes the EMT occurrence with E‐cadherin low‐expression in glioblastoma.^10^ In addition, the activation of TGF‐β1/Smad signalling is detected in cirrhotic liver of patients^30^ and carbon monoxide‐induced liver fibrosis of mice.^5^ In this study, fructose‐induced survivin over‐expression and TGF‐β1/Smad signalling activation were also observed in rat livers and BRL‐3A cells. Furthermore, we found that fructose‐induced survivin over‐expression provoked the activation of TGF‐β1/Smad signalling to develop the EMT, causing liver fibrosis. Therefore, we focused on the regulation of survivin in fructose‐induced EMT in liver fibrosis. In relation to this, it is worth noting that miR‐203 low‐expression decreases E‐cadherin in ovarian cancer cells line through a survivin‐dependent manner.^12^ We observed miR‐203 low‐expression in the animal and cell models, being consistent with these reports in carbon tetrachloride‐induced fibrotic liver of rodents,^26^ arecoline‐induced fibrotic oral submucous of patients^31^ and cirrhotic livers of patients.^32^ We also showed that survivin was a target gene of miR‐203 in BRL‐3A cells. Importantly, miR‐203 up‐regulation nearly abrogated survivin over‐expression in fructose‐exposed BRL‐3A cells. These results suggest that fructose‐induced miR‐203 low‐expression may target survivin to activate TGF‐β1/Smad signalling, causing the EMT.

Transcription factor ZEB1 suppresses miR‐203 expression and then activates cancer cell epithelial differentiation.^14^ High ZEB1 expression is observed in hepatocellular carcinoma patients with or without cirrhosis.^33^ In this study, we found that fructose increased nuclear ZEB1 protein levels in the animal and cell models. This fructose‐induced ZEB1 change was earlier than miR‐203 low expression in BRL‐3A cells. Indeed, *ZEB1* siRNA abrogated fructose‐caused miR‐203 low expression in BRL‐3A cells. These results provide the solid evidence that fructose‐induced ZEB1 nuclear translocation causes miR‐203 low expression, which may be a suitable alternative for targeting survivin to activate TGF‐β1/Smad signalling in fructose‐driven EMT and liver fibrosis. This new finding has not been previously explored, to our knowledge. ZEB1 nuclear translocation triggered by fructose, as a switch, may suppress miR‐203 expression to cause the EMT in liver fibrosis (Figure 5J). Thus, ZEB1 nuclear translocation inhibition with high miR‐203 expression may be a predictor of good prognosis in patients with liver fibrosis. Of note, phosphorylation of signal transducer and activator of transcription 3 (STAT3) can translocate into the nucleus to recruit on ZEB1 promoter, resulting in ZEB1 nuclear translocation in breast cancer cells.^34^ Our previous study found that fructose up‐regulated p‐STAT3 in rat liver fibrosis.^35^ Therefore, we speculated that fructose‐induced up‐regulation of p‐STAT3 may transfer into the nucleus to recruit on ZEB1 promoter, causing ZEB1 nuclear translocation in rat liver fibrosis. However, the precise molecular mechanism by which fructose induces ZEB1 nuclear translocation needs further study.

Polydatin is reported to down‐regulate TGF‐β1 in unilateral ureter obstruction‐induced fibrotic kidney of rats^36^ and radiation‐induced fibrotic lung of mice.^22^ It also inhibits TGF‐β1 and collagen, reduces TGF‐β1‐induced EMT in human alveolar epithelium A549 cells^37^ and protects against methionine‐choline deficient diet‐induced mouse liver fibrosis.^21^ Pioglitazone decreases hepatic TGF‐β1 and COL1A1 in non‐alcoholic steatohepatitis of mice.^38^, ^39^ In this study, polydatin and pioglitazone effectively down‐regulated survivin to inhibit the activation of TGF‐β1/Smad signalling and repress the EMT, preventing against fructose‐caused liver fibrosis. Furthermore, polydatin and pioglitazone increased miR‐203 expression in vivo and in vitro under fructose condition. These effects further inhibited survivin to block TGF‐β1/Smad signalling activation in the attenuation of fructose‐induced EMT in liver fibrosis. More importantly, polydatin and pioglitazone suppressed ZEB1 nuclear translocation to up‐regulate miR‐203 expression, and then decreased survivin to suppress TGF‐β1/Smad signalling activation in the reduction of the EMT (Figure 5J). Therefore, the ability of polydatin and pioglitazone to inhibit ZEB1 nuclear translocation and increase miR‐203 expression is the key to attenuate the EMT in the protection against liver fibrosis associated with high fructose intake through the blockade of survivin‐mediated TGF‐β1/Smad signalling activation.

In conclusion, this study demonstrates that fructose causes ZEB1 nuclear translocation to decrease miR‐203 expression, and then target survivin to activate TGF‐β1/Smad signalling, developing the EMT in liver fibrosis. ZEB1 nuclear translocation inhibition with high miR‐203 expression may be the predictor of good prognosis in patients with liver fibrosis. Polydatin protects against fructose‐induced hepatocyte EMT by suppressing ZEB1 nuclear translocation to up‐regulate miR‐203 expression and block survivin‐activated TGF‐β1/Smad signalling, exhibiting the potential anti‐liver fibrosis activity. The present study also supports that the blockade of ZEB1 nuclear translocation by polydatin is a novel strategy for attenuating EMT in liver fibrosis associated with high fructose consumption.

## CONFLICT OF INTEREST

The authors confirm that there are no conflicts of interest.

## AUTHOR CONTRIBUTION

**Ling‐Dong Kong:** Funding acquisition (lead); Investigation (lead); Methodology (supporting); Project administration (lead); Software (supporting); Writing‐original draft (lead); Writing‐review & editing (lead). **Xiao‐Juan Zhao:** Data curation (lead); Formal analysis (lead); Investigation (lead); Methodology (lead); Project administration (supporting); Software (lead); Writing‐original draft (lead); Writing‐review & editing (lead). **Yan‐Zi Yang:** Investigation (supporting); Methodology (supporting); Software (supporting). **Han‐Wen Yu:** Investigation (supporting); Methodology (supporting); Software (supporting). **Wen‐Yuan Wu:** Investigation (supporting); Methodology (supporting); Software (supporting). **Yang Sun:** Formal analysis (equal); Investigation (lead); Methodology (lead). **Ying Pan:** Formal analysis (equal); Investigation (supporting); Methodology (supporting); Project administration (lead); Writing‐original draft (lead); Writing‐review & editing (lead).

Lingdong Kong: Conception of study. Lingdong Kong, Xiaojuan Zhao and Yang Sun: Experiments design. Xiaojuan Zhao: Carry out animal and cell experiments. Yanzi Yang: Help animal experiments. Ying Pan and Hanwen Yu: Help cell culture and immunofluorescence experiments. Wenyuan Wu: Help dual‐luciferase reporter assay. Xiaojuan Zhao, Ying Pan and Lingdong Kong: Data analysis. Lingdong Kong, Xiaojuan Zhao and Ying Pan: Manuscript writing and manuscript editing.

## Data Availability

The data that support the finding of this study are available from the corresponding author upon reasonable request.
